# Typology and Ethical Considerations of Digital Health Promotion Tools for Youth in Sub-Saharan Africa: Review of Examples From Ghana, Kenya, and South Africa

**DOI:** 10.2196/54472

**Published:** 2025-09-15

**Authors:** Agata Ferretti, Shannon Hubbs, Richard Mawutor Dzikunu, Keymanthri Moodley, Frederick Murunga Wekesah, Jonty Wright, Effy Vayena

**Affiliations:** 1 Department of Health Sciences and Technology, ETH Zurich, Hottinherstrasse 10, Zurich, 8092, Switzerland; 2 YIELD Hub, Rutgers International, Utrecht, The Netherlands; 3 Division for Medical Ethics and Law, Faculty of Medicine and Health Sciences, Stellenbosch University, Western Cape, South Africa; 4 African Population and Health Research Center, Nairobi, Kenya; 5 Faculty of Medicine and Health Sciences, Stellenbosch University, Western Cape, South Africa

**Keywords:** youth, young people, low- and middle-income countries, LMICs, health promotion, digital health, Sub-Saharan Africa, taxonomy, digital health, tool, African, technology, digital tool, health information, digital health information

## Abstract

**Background:**

Digital technologies for health promotion have proliferated over the past decade, with uptake increasing steadily among young people, including those in low- and middle-income countries (LMICs). Youth increasingly rely on digital tools for health information, and the early influence of this digital technology can have an impact throughout the lifespan. While there is a growing body of literature on the opportunities and challenges of digital health promotion (DHP) for young people, a gap remains in research that closely examines the characteristics of digital health strategies developed specifically for youth in LMICs.

**Objective:**

In this paper, we investigate and compare selected examples of DHP tools from 3 countries in Sub-Saharan Africa, namely Ghana, Kenya, and South Africa. Our aim is to create a multidimensional descriptive typology of DHP tools developed specifically to promote the health of adolescents and young adults in these countries.

**Methods:**

To select the tools, we conducted systematic internet-based searches using relevant keywords, incorporating the expertise of local professionals to ensure a thorough search. Included solutions originated from one of the 3 countries of focus and could take any number of forms such as apps, websites, chatbots, or social media initiatives. We thereafter deductively created a typology describing selected features of each tool, including the health area of focus, key stakeholders, type of service, and ethical values explicitly referenced within the tool. While such high-level features of interest were selected based on the existing literature in the field, the detailed descriptive categories were identified through an inductive analysis of the tools.

**Results:**

A total of 31 DHP tools were identified. Sexual and reproductive health was the most common health area of focus for DHP services, which were primarily funded and supported by local non-governmental organizations, foundations, and international organizations. The assessed tools were predominantly web-based and social media-based, with the overarching goal and core value of expanding health knowledge and offering access to health promotion services to young people.

**Conclusions:**

With sustained investment, DHP can improve the health of young people while relieving pressure on health care services. The areas of mental health, as well as substance use prevention and nutrition, stand out with clear potential for health gains through investment in DHP. Addressing ethical concerns such as privacy, transparency, equity, and inclusiveness is essential to the safety, usefulness, and fairness of DHP. To achieve the greatest benefit, local youth perspectives and priorities should be included in DHP development. Local initiatives have the potential to be the most agile, flexible, and relevant for the target audience of young people, with the overall goal of early intervention and greater health quality throughout the lifespan, and more efficient use of health care resources.

## Introduction

The use of technology to promote the health of youth is gaining popularity due to the increasing availability of digital tools worldwide. Three-quarters of the world’s population owns a smartphone, and in 2022, 66% of the world’s population had access to the internet [1]. In low- and middle-income countries (LMICs), the uptake of digital technologies increased over the past decade, offering marginalized and underresourced population groups the opportunity to access services (eg, health care) remotely [2].

Young individuals are among those most engaged with technology, increasingly turning to the internet, adopting digital tools, and asking questions to artificial intelligence–powered interfaces. While rates and reliability of access vary significantly between and within individual countries, recent data indicate that internet accessibility among young people in LMICs stands at approximately 67% and reaches 95% in upper-middle-income economies [3]. However, it is important to note that a reliable and consistent internet connection, as well as access to personal devices, remains a challenge due to infrastructural limitations and socioeconomic disparities [4].

Youth tend to rely on technology (such as apps, social media, podcasts, websites, and forums) to obtain health-related information and to be supported in maintaining a healthy lifestyle [5]. Such use of digital technology by youth can contribute to the mitigation of health risk factors and ultimately reduce the burden of diseases [6,7]. Notably, adolescence and young adulthood, as defined by the World Health Organization to include people of the ages 10‐24, represent a pivotal period for health promotion interventions [8]. Such interventions can positively impact lifelong health by modifying lifestyle habits and raising awareness about health topics during this transitional phase [9]. Novel digital apps can encourage new possibilities for awareness of one’s own health and create new means of caring for one’s personal health needs [10].

Effective digital health promotion (DHP) for youth is one valuable means of intervention to combat health inequalities that can emerge early in life, and whose effects can persist throughout the lifespan [11,12]. Although for young people, health inequalities are often related to social inequalities and factors such as family income, stability of parent employment, housing, or ethnicity, active engagement in healthy behaviors can play a role in improving and maintaining well-being [13]. DHP holds potential to positively impact young people in LMICs in particular, as in these regions, technology offers one solution to overcome the limitations of public health systems that grapple with resource constraints. Digital means can reach youth, irrespective of their physical proximity to health care facilities, the presence of national health campaigns, or the availability of health practitioners, thereby not adding to the burden on these systems.

In 2017, Ippoliti and L’Engle [14] reviewed health projects in LMICs that used mobile phones as a means for improving adolescent health, noting that the appeal of mobile technology for health apps lies partly in its affordability and confidentiality. Health promotion was the purpose of a majority of the examined initiatives, which used such methods as text messaging, hotlines, and social media. While this work did not specifically address ethical considerations of mobile health technology for adolescents, it is an example of an earlier effort to investigate and describe available digital health promotion tools for young people in LMICs.

Despite the growing availability of literature on the opportunities and challenges of DHP for young people [15,16], research closely examining the characteristics of DHP strategies specifically developed for and in LMICs and targeting youth in these countries is still missing. Therefore, the objective of this paper is to identify existing DHP strategies that have been developed by different entities for youth in sub-Saharan Africa (SSA), and to establish a typology for these tools, including highlights of their key features. We focused our investigation on 3 English-speaking countries in SSA: Ghana, Kenya, and South Africa.

In these 3 countries, youth face significant challenges to their well-being, including a high burden of HIV and sexually transmitted diseases, road injuries, interpersonal violence, malnutrition, and mental health conditions [8]. Primary risk factors affecting this demographic encompass unsafe sexual practices, limited knowledge of sexual and mental health, unaddressed existing mental health conditions, insufficient physical activity, unhealthy dietary habits, overweight concerns, and substance use (smoking, binge drinking, and drug usage) [17]. These areas of health present opportunities for intervention through enhanced health awareness and individualized reinforcement of positive health behavior strategies, which stand at the core of DHP.

## Methods

### Overview

In May 2023, we aimed to identify examples of active digital health promotion tools and strategies targeting youth in the 3 countries of interest (Ghana, Kenya, and South Africa). Given the absence of a centralized repository for DHP tools, we identified initiatives through systematic online searches using English-language keywords related to youth, health promotion, and digital media. In addition, to ensure a comprehensive survey of available tools, we relied on the expertise of local professionals in each of the included countries (RMD, FW, JW, and KM) who work in the field of youth health and digital technologies and were knowledgeable about relevant examples. The list of tools we compiled reflects the specific moment in time when the search was conducted and will likely evolve as new tools emerge and others become inactive or unavailable. However, this represents the first effort in this direction to collect and analyze digital health promotion tools for youth in SSA, with the aim that future research will build upon.

To be included in our study, DHP initiatives needed to explicitly focus on young people, adolescents, or young adults as the primary users or stakeholders. Furthermore, they should originate from one of the 3 countries of interest and be developed and sponsored by local non-governmental organizations (NGOs), foundations, entrepreneurs, private sector entities, research institutions, or governments. Therefore, we did not consider apps and strategies that were created in other parts of the world, or that were introduced and promoted by global brands or multinational corporations, in order to maintain a regional focus. Included DHP tools took various forms, such as websites, social media interventions, texting services (eg, WhatsApp and chatbots), mobile health and fitness apps, and audio services (eg, podcasts and calls) [18].

Once the tools were identified, we established a typology based on deductively selected descriptive variables. We derived these variables from existing literature on the subject, emphasizing key characteristics of DHP [18-22]. Specifically, we examined the tool’s health focus area, the key stakeholders and actors involved in its development and deployment, the format and type of services provided, and the ethical principles and values explicitly promoted within the DHP or in its content. The subcategories within each feature were identified inductively. Two researchers independently reviewed the online content of each tool, comparing their analyses and discussing any disagreements to reach a consensus. Importantly, we only analyzed primary information available directly on the websites of the tools and did not use secondary data about these tools (eg, articles published about such tools).

In compiling and describing various typologies of DHP in Ghana, Kenya, and South Africa, we acknowledge that our efforts do not encompass all available tools, as some may not have been detected in our search, and as new tools are continuously being developed. Nonetheless, we aimed to maintain a systematic approach.

It is important to note that inclusion of these tools in our analysis does not imply endorsement of these specific initiatives and products. Rather, we would like to showcase examples of existing DHP services that are presently available to any young user, and to report on the variety of features offered by these tools.

### Ethical Considerations

Data used in our study consisted of information that is publicly available on digital health promotion initiatives online. Therefore, no anonymization or deidentification was carried out in the course of this study.

## Results

### Overview

**Figure 1.**
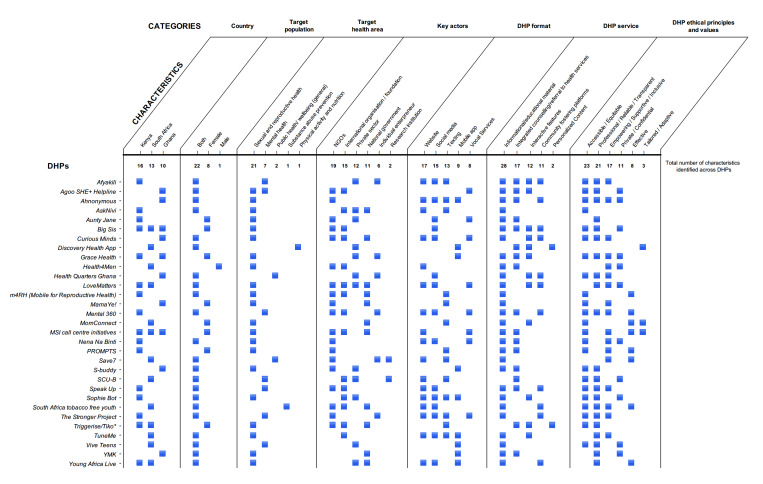
Typology of examples of digital health promotion tools for young people from Ghana, Kenya, and South Africa, as identified via systematic online search in May 2023. Search criteria included English-language keywords related to youth, health promotion, and digital media, with additional consultation with local professionals to gather any further relevant tools available at that time. Analyzed characteristics include country, target population, target health area, key actors, format, service, and ethical principles and values. DHP: digital health promotion.

We identified a total of 31 DHP tools, including 16 in Kenya, 13 in South Africa, and 10 in Ghana (Figure 1). All tools originated in one of the 3 countries, with some tools available in multiple countries. The majority of these tools were designed for all young users, with few designed specifically for women, and notably, only 1 out of 31 targeting men.

### DHP Area of Health Focus

The majority of DHP tools concentrated on sexual and reproductive health topics (21/31), which is understandable given the prevalence of underage pregnancies, HIV, and other sexually transmitted infections in the 3 countries, and the associated maternal health risks. We found a limited number of DHP solutions addressing youth mental health (7/31), an area that is steadily gaining attention in the region. Notably, we identified a scarcity of locally developed DHP targeting substance abuse prevention or promoting healthy lifestyle habits [23].

### Key Stakeholders and Actors Involved in DHP Development and Deployment

The development and promotion of most DHPs was facilitated by local NGOs (19/31) and foundations and international organizations (15/31), with the private sector (eg, health insurance and tech companies) also offering substantial support (12/31). National governments also contributed to the development of existing DHPs (11/31), supporting broad initiatives often made available at the regional or country level. The role of individual entrepreneurs (6/31) and research institutions (2/31) was relatively minor among the examples we identified.

### DHP Formats and Type of Services Provided

The majority of the identified DHP tools were primarily accessible through websites (17/31), followed by availability on social media platforms (15/31) and texting solutions, including messaging and chatbots (9/31). Only a small number of DHP solutions consisted of dedicated mobile apps (9/31).

The majority of the reviewed DHP solutions (28/31) offered informational and educational materials to users. In addition, integrated counseling services, the option to engage with experts, and referrals to health services were provided by 17 out of 31 DHP services. This finding appears to be in contrast with what young people have commonly cited as an appeal of DHP, namely, the possibility to avoid discussions with biased adults or health care professionals regarding sensitive or potentially taboo topics [18]. However, the inclusion of such services in countries such as Ghana, Kenya, and South Africa can potentially be explained by the cultural value of involving others in decision-making and seeking the input of medical professionals [4].

Interactive design (12/31), platforms fostering community interaction (11/31), and personalized content (2/31) were less commonly featured in the analyzed tools. This aspect is noteworthy, as it seems to conflict with what youth report to be the most important DHP features. Research shows that young people tend to engage more and stay committed to using digital solutions when they find them aesthetically appealing and functional [24]. They also appreciate content tailored specifically to their needs [25,26]. Moreover, young people value opportunities to share their concerns with peers and receive feedback from those who have faced similar experiences, as such peer dynamics enable a sense of support and understanding [22].

### Ethical Principles and Values Explicitly Endorsed by DHP

DHP tools frequently emphasized ethical values and considerations among their stated goals and missions. Most often cited (23/31) was increasing accessibility of health information and health promotion services, thereby expanding opportunities to a greater number of youths and complementing the work of traditional health systems. Low-budget (if not in most cases, free) DHP strategies are presented as a solution for young people wishing to take care of their own health, reaching them via digital tools regardless of socioeconomic, cultural, or location barriers. In addition, by leveraging design and aesthetics, as well as audio and visual features, these health promotion strategies engage young people at all levels of education and health literacy, making DHP a more equitable tool.

Many of the DHP initiatives analyzed also emphasized values of transparency, truthfulness, and reliability of the information provided to users (21/31). In particular, these values are realized by combating data misinterpretation and misinformation. This is accomplished by providing users with scientifically valid and verifiable information that is supported by sound sources of data, integrating the ability to contact health experts on demand, combating fake news, and being transparent about which data will be used by the DHP tool. Inclusiveness and empowerment also recurred frequently in ethics and value statements (17/31). Notably, DHP emphasizes being user-centered (youth-focused and often youth-driven), supporting individuals (and indirectly communities) in behaviors that will help their health and specific well-being to flourish, and including all individuals, rejecting stigma or cultural barriers.

In contrast, ethical issues related to privacy (11/31), effectiveness (8/31), and adaptability of DHP featured less among the mission and value statements of the DHP tools we identified.

## Discussion

### Principal Findings and Comparison With Previous Works

Our analysis and creation of a multidimensional typology of DHP strategies identifies recurrent features, as well as potential gaps and areas for further development, among identified active DHP tools in Ghana, Kenya, and South Africa. Youth in these countries face a variety of unique health challenges and risks. According to our results, presently available solutions offer a great deal to young people as health promotion resources, but do not fully address these health needs, suggesting a significant untapped opportunity for investment in DHP. Our results indicate that this is particularly true in the area of youth mental health, as well as for substance abuse prevention and nutrition. Apps developed in Western contexts frequently cover these topics, but may be less relevant for the specific health needs of youth in SSA. For example, DHP apps that recommend outdoor physical activity or specific dietary habits may be impractical in areas where it is unsafe to be outside or where food options are limited. Thus, DHP developed locally with input from health experts can enrich the landscape of fit-for-the-setting solutions.

Our findings demonstrate that many DHP initiatives available to youth in SSA are already formed and operate locally, with agile development processes that are not reliant on bureaucratic systems or formal government programs for approval. Such smaller-scale projects run by regional NGOs offer flexibility in tailoring tools and content to the needs of specific youth populations and vulnerable groups. Such solutions can not only take into account local barriers to the implementation of DHP, but can also integrate local values, such as relational privacy or group solidarity. This agility, together with the potential for valuable local data collection, provides further rationale for private sector investment in DHP.

Our findings suggest that currently available DHP tools may only partially address the health needs and risk factors most prevalent among youth in these 3 SSA countries, for example, regarding substance abuse prevention, mental health, and nutrition. This omission could be attributed to factors such as potential stigma around mental health and substance use issues, and their absence from cultural dialogue (hindering investment or cultural support); greater incentive to invest in DHP strategies for issues considered more critical (eg, HIV); or lack of data and understanding of the significance of these areas of adolescent health, among developers or government bodies. In our survey of DHP tools, only one aimed to address more than one area of health. This could be due to the need to be effective and valuable to users while standing out in a competitive market. To succeed, tools must offer content tailored to the needs of youth, and therefore diversifying across multiple health areas could be counterproductive, diluting their focus and reducing their impact.

Our data highlight the prevailing preference in LMICs for cost-effective solutions such as websites, with developers and investors often opting to leverage existing infrastructure such as social media and texting platforms, instead of developing entirely new apps that demand additional resources. In addition, if the youth population is receptive to tools developed in Western countries and sponsored by large companies, it can be more challenging for local providers and developers to secure market space.

DHP presents an opportunity to intervene early in unhealthy behaviors, thereby fostering health and reducing the number of individuals who may require acute or chronic care as adults. DHP encourages individuals to take an active role in managing their own health rather than being passive recipients of medical advice. These factors make a strong case for these national governments to continue to invest in DHP projects. Supporting tools that strengthen health education and promote healthy behavior can lead to long-term benefits, ultimately reducing the burden on health care systems. Involving multiple investors with reliable funding can enhance the sustainability of DHP programs beyond one-time strategies and short-term campaigns.

Interdisciplinary teams of experts and community leaders who focus on technical aspects and also ensure ethical alignment of DHP could be valuable. This analysis confirms that crucial ethical issues such as individual and group privacy, equity, inclusiveness, and transparency should not be overlooked, especially in settings where access to health care and health literacy is limited. While our analysis suggests a strong focus among tool developers on the reliability of health information, the literature points to the frequent inaccuracy of information shared by DHP tools. For the time being, beyond checks instituted by developers themselves, there are no independent review mechanisms assessing information provided to users. In the absence of external oversight, a holistic approach to the development and implementation of DHP, beginning with interdisciplinary teams, can mitigate the dissemination of unreliable and potentially harmful information to users. Referencing sources, being transparent about the criteria used to develop recommendations, introducing feedback systems, and developing open-source digital health software repositories are further steps to ensure that DHP solutions are more ethically aligned and safe.

Reflecting on the ethical principles specifically endorsed by DHP tools, research indicates that privacy and effectiveness are not among young people’s top priorities when considering DHP, which may in turn lead developers and deployers not to prioritize these values [16,18,27]. In SSA contexts, Western ideas of privacy and technology tailored to individual needs give way to more fundamental interpretations based on relational aspects and group dynamics. However, glossing over these values could signal inadequate recognition of their significance. Further reflection is warranted concerning implications for privacy breaches, tool effectiveness, and tailoring content to user needs.

As new DHP initiatives are developed regularly, our review captures the tools that the authors were able to identify at the time of our search. Thus, our analysis examines a selection of DHP tools that is not comprehensive. While 2 reviewers examined all information accessible online about each tool and reached agreement in our findings, our analysis was limited to the data currently provided about each initiative.

This analysis points to areas of misalignment between the formats and features of DHP tools and the preferences of young people as described in the literature, raising questions about the process of conception and development of initiatives intended for young people. It suggests that if youth have more meaningful involvement in developing DHP tools for their use, the result will be even better suited to their needs. Our analysis also demonstrates that existing literature reports predominantly on research in high-income countries, reflecting the perspectives of youth in these countries. This ambiguity underscores the need for greater engagement and meaningful consultation with youth in LMICs, in planning and developing DHP tools, and in research on digital health solutions.

### Conclusions

This paper presents and compares selected examples of digital tools and solutions for youth health promotion in SSA, particularly in Ghana, Kenya, and South Africa. As DHP becomes increasingly available to youth, it is important to analyze their characteristics to better understand the strengths and pitfalls present in different settings. Our work to create a typology of DHP characteristics in these countries aims to highlight recurring patterns among the tools, as well as possible gaps that should be addressed to ensure ethically aligned technologies. In addition to expanding access to DHP in LMICs by increasing investment in the health sector and empowering local developers to create context-specific digital programs, our analysis shows the need to incorporate a number of ethical principles in the development and deployment of such tools. Ethical considerations of inclusiveness, equity, transparency, effectiveness, and personalization cannot be neglected, if not to the detriment of the quality and safety of DHP. Only by meaningfully engaging young people and knowing their concerns is it possible to create tools that effectively meet users’ needs and that are sustainable over time. As research on youth perspectives on digital health progresses, technology development should adapt and incorporate such knowledge and feedback.

## Abbreviations

DHP: digital health promotion
LMIC: low- and middle-income countries
NGO: non-governmental organization
SSA: sub-Saharan Africa
